# Correction: A brain-specific PGC1a fusion transcript affects gene expression and behavioral outcomes in mice

**DOI:** 10.26508/lsa.202101295

**Published:** 2021-11-24

**Authors:** Oswaldo A Lozoya, Fuhua Xu, Dagoberto Grenet, Tianyuan Wang, Korey D Stevanovic, Jesse D Cushman, Thomas B Hagler, Artiom Gruzdev, Patricia Jensen, Bairon Hernandez, Gonzalo Riadi, Sheryl S Moy, Janine H Santos, Richard P Woychik

**Affiliations:** 1 Genomic Integrity and Structural Biology Laboratory, National Institutes of Health, Durham, NC, USA; 2 Integrative Bioinformatics Branch, National Institutes of Health, Durham, NC, USA; 3 Neurobehavioral Core Laboratory, National Institutes of Health, Durham, NC, USA; 4 Knockout Mouse Core Facility, National Institutes of Health, Durham, NC, USA; 5 Neurobiology Laboratory, National Institute of Environmental Health Sciences, National Institutes of Health, Durham, NC, USA; 6 Centro de Bioinformática y Simulación Molecular, Facultad de Ingeniería, Universidad de Talca, Talca, Chile; 7 Department of Psychiatry, Carolina Institute for Developmental Disabilities, University of North Carolina at Chapel Hill, Chapel Hill, NC, USA

It came to our attention that the Gene Chip utilized for microarray analysis wrongly indicated the Human GeneChip. Instead, the microarrays used the Affymetrix Mouse GeneChip as below. We apologize for the inconvenience; this correction bears no impact on the microarray results obtained.

Article: Lozoya OA, Xu F, Grenet D, Wang T, Stevanovic KD, Cushman JD, Hagler TB, Gruzdev A, Jensen P, Hernandez B, Riadi G, Moy SS, Santos JH, Woychik RP (2021 Oct 14). A brain-specific pgc1α fusion transcript affects gene expression and behavioral outcomes in mice. Life Sci Alliance 4(12):e202101122. doi: 10.26508/lsa.202101122. PMID: 34649938; PMCID: PMC8548212.

Where it reads:

**Microarrays, qRT-PCR, and data analysis.** The *Affymetrix Human Genome U133 Plus 2.0 GeneChip* arrays were used to profile gene expression. Samples were prepared as per manufacturer’s instructions. Arrays were scanned in an Affymetrix Scanner 3000 and data was obtained using the GeneChip Command Console and Expression Console Software (AGCC; Version 3.2 and Expression Console; Version 1.2) using the MAS5 algorithm to generate CHP-extension files.

It should read:

**Microarrays, qRT-PCR, and data analysis.** The *Affymetrix Mouse Genome 430 2.0 GeneChip* arrays were used to profile gene expression. Samples were prepared as per the manufacturer’s instructions. Arrays were scanned in an Affymetrix Scanner 3000 and data was obtained using the GeneChip Command Console and Expression Console Software (AGCC; Version 3.2 and Expression Console; Version 1.2) using the MAS5 algorithm to generate CHP-extension files.

## Supplementary Material

Reviewer comments

