# Supplementary material for: Correction: A brain-specific PGC1a fusion transcript affects gene expression and behavioral outcomes in mice
Source: Life Sci Alliance. 2021 Nov 24;5(2):e202101295. doi: 10.26508/lsa.202101295 (PMC8616557; doi:10.26508/lsa.202101295)
Supplement: Reviewer comments [file LSA-2021-01295_review_history.pdf]

Life Science Alliance

Correction:A brain specific PGC1a fusion transcript affects gene express. & behav. outcomes in mice

Janine Santos, Oswaldo Lozoya, Fuhua Xu, Dagoberto Grenet, Tianyuan Wang, Korey Stevanovic, Jesse Cushman, Thomas Hagler, Artiom Gruzdev, Patricia Jensen, Bairon Hernandez, Gonzalo Riadi, Sheryl Moy, and Richard Woychik
DOI: https://doi.org/10.26508/lsa.202101295

Corresponding author(s): Janine Santos, National Institute of Environmental Health Sciences and Richard Woychik, NIEHS

Review Timeline:

Submission Date:	2021-11-10
Accepted:	2021-11-12

Scientific Editor: Eric Sawey, PhD

Transaction Report:

(Note: With the exception of the correction of typographical or spelling errors that could be a source of ambiguity, letters and reports are not edited. The original formatting of letters and referee reports may not be reflected in this compilation.)

November 12, 2021

RE: Life Science Alliance Manuscript #LSA-2021-01295

Dr. Janine H. Santos
National Institute of Environmental Health Sciences
111 TW Alexander drive
Durham, NC 27709

Dear Dr. Santos,

Thank you for submitting your Correction entitled "Correction:A brain specific PGC1a fusion transcript affects gene express. & behav. outcomes in mice".

You can contact the journal office with any questions, contact@life-science-alliance.org

Sincerely,

Eric Sawey, PhD
Executive Editor
Life Science Alliance
http://www.lsajournal.org

November 12, 2021

RE: Life Science Alliance Manuscript #LSA-2021-01295

Dr. Janine H. Santos
National Institute of Environmental Health Sciences
111 TW Alexander drive
Durham, NC 27709

Dear Dr. Santos,

Thank you for submitting your Correction entitled "Correction:A brain specific PGC1a fusion transcript affects gene express. & behav. outcomes in mice".

You can contact the journal office with any questions, contact@life-science-alliance.org

Sincerely,

Eric Sawey, PhD
Executive Editor
Life Science Alliance
http://www.lsajournal.org
